# Detection of chronic wasting disease in mule and white-tailed deer by RT-QuIC analysis of outer ear

**DOI:** 10.1038/s41598-021-87295-8

**Published:** 2021-04-08

**Authors:** Natalia C. Ferreira, Jorge M. Charco, Jakob Plagenz, Christina D. Orru, Nathanial D. Denkers, Michael A. Metrick, Andrew G. Hughson, Karen A. Griffin, Brent Race, Edward A. Hoover, Joaquín Castilla, Tracy A. Nichols, Michael W. Miller, Byron Caughey

**Affiliations:** 1 Laboratory of Persistent Viral Diseases, Rocky Mountain Laboratories, National Institute of Allergy and Infectious Diseases, National Institutes of Health, Hamilton, MT USA; 2 CIC bioGUNE, Basque Research and Technology Alliance (BRTA), Bizkaia Technology Park, Derio, Spain; 3 Prion Research Center, Department of Microbiology, Immunology, and Pathology, College of Veterinary Medicine and Biomedical Sciences, Colorado State University, Fort Collins, CO USA; 4 Colorado Division of Parks and Wildlife, Wildlife Health Program, 4330 Laporte Avenue, Fort Collins, CO USA; 5 IKERBASQUE, Basque Foundation for Science, Bilbao, Spain; 6 USDA-APHIS-VS-Cervid Health Program, Fort Collins, CO USA

**Keywords:** Biological techniques, Microbiology, Biomarkers, Diseases of the nervous system, Diseases, Infectious diseases, Neurological disorders

## Abstract

Efforts to contain the spread of chronic wasting disease (CWD), a fatal, contagious prion disease of cervids, would be aided by the availability of additional diagnostic tools. RT-QuIC assays allow ultrasensitive detection of prion seeds in a wide variety of cervid tissues, fluids and excreta. The best documented *antemortem* diagnostic test involving RT-QuIC analysis targets lymphoid tissue in rectal biopsies. Here we have tested a more easily accessed specimen, ear pinna punches, using an improved RT-QuIC assay involving iron oxide magnetic extraction to detect CWD infections in asymptomatic mule and white-tailed deer. Comparison of multiple parts of the ear pinna indicated that a central punch spanning the auricular nerve provided the most consistent detection of CWD infection. When compared to results obtained from gold-standard retropharyngeal lymph node specimens, our RT-QuIC analyses of ear samples provided apparent diagnostic sensitivity (81%) and specificity (91%) that rivaled, or improved upon, those observed in previous analyses of rectal biopsies using RT-QuIC. These results provide evidence that RT-QuIC analysis of ear pinna punches may be a useful approach to detecting CWD infections in cervids.

## Introduction

Chronic wasting disease (CWD) is a fatal, and contagious prion disease of cervids^1^. CWD is characterized by a prolonged incubation period, which can vary from 2 to 4 years^2^, after which animals develop clinical disease and die^3^. The long incubation period contributes to transmission as asymptomatic animals shed infectious prions in excreta and biological fluids^4–7^. Susceptible animals can be infected directly, through the contact with saliva, urine, feces or aerosols from an infected animal; or indirectly (environmental contamination), due to the ingestion of infectious prions bound to soil or plants, for instance^4,8^. CWD is characterized by the accumulation of the pathogenic form of prion protein, termed PrP^Sc^. Following oral ingestion, the spread of PrP^Sc^ throughout the body follows a specific pattern, being first identified in the peripheral lymphoreticular system and later in the central nervous system^9–11^. Because retropharyngeal lymph nodes (RLN) are one of the first sites of prion replication, this tissue, along with a specific portion of the medulla oblongata (the obex), are the gold standard tissues approved for CWD detection by immunohistochemistry and ELISA (http://www.aphis.usda.gov/animal_health/animal_diseases/cwd/downloads/cwd-program-standards.pdf) (for review, see^12^). However, CWD surveillance and control in wildlife would be aided by the availability of accurate tests applicable to more accessible specimens that could be easily collected from live, as well as dead, cervids.

Toward this goal, ultrasensitive seed amplification assays^13^, such as real-time quaking-induced conversion (RT-QuIC)^14,15^, and protein misfolding cyclic amplification (PMCA)^16^, have been shown to be capable of detecting minute amounts of prions in CWD-infected animals through the analysis of certain peripheral tissues, biological fluids, and excreta^4–7,15,17–21^. In RT-QuIC reactions, PrP^Sc^ that may be present in a sample seeds the refolding and polymerization of the monomeric recombinant PrP^C^ (the substrate), which is in vast excess in the reaction mix^13^. The resulting amyloid fibrils enhance the fluorescence of thioflavin T which is used to monitor the polymerization process over time. Cycles of shaking and rest accelerate the seeded polymerization reaction. Seed amplifications of a billion-fold or more can be achieved in RT-QuIC assays. Of particular interest with respect to *antemortem* diagnosis are multiple studies involving hundreds of white-tailed deer^22^ or elk^23,24^ showing that RT-QuIC analysis of rectal biopsy specimens yielded 65–83% sensitivity and 94–100% specificity. These RT-QuIC sensitivities were substantially higher than those obtained using immunohistochemistry for abnormal PrP deposits in recto-anal mucosa associated lymphoid tissue (RAMALT). Nonetheless, further improvements in diagnostic sensitivity, specificity, and practicality of antemortem CWD tests would facilitate the monitoring of CWD in cervids.

Previous studies have shown the accumulation of readily detectable prion seeding activity in the skin of humans with Creutzfeldt–Jakob disease^25,26^, and rodents with scrapie, even early in the course of infection^27^. Given that the outer ear could serve as an accessible source of both skin and nervous tissue, we sought to investigate the utility of RT-QuIC to diagnose CWD in pre-clinical white-tailed and mule deer using ear pinna punches. Here, by coupling iron oxide magnetic extraction to a modified RT-QuIC assay, we were able to diagnose CWD by analyzing ear samples collected from both clinical and asymptomatic animals with an accuracy at least as good as previous assays of RAMALT tissue, with the advantage herein of using a more easily collectable specimen.

## Results

### RT-QuIC analysis of RLN samples

**Figure 1:**
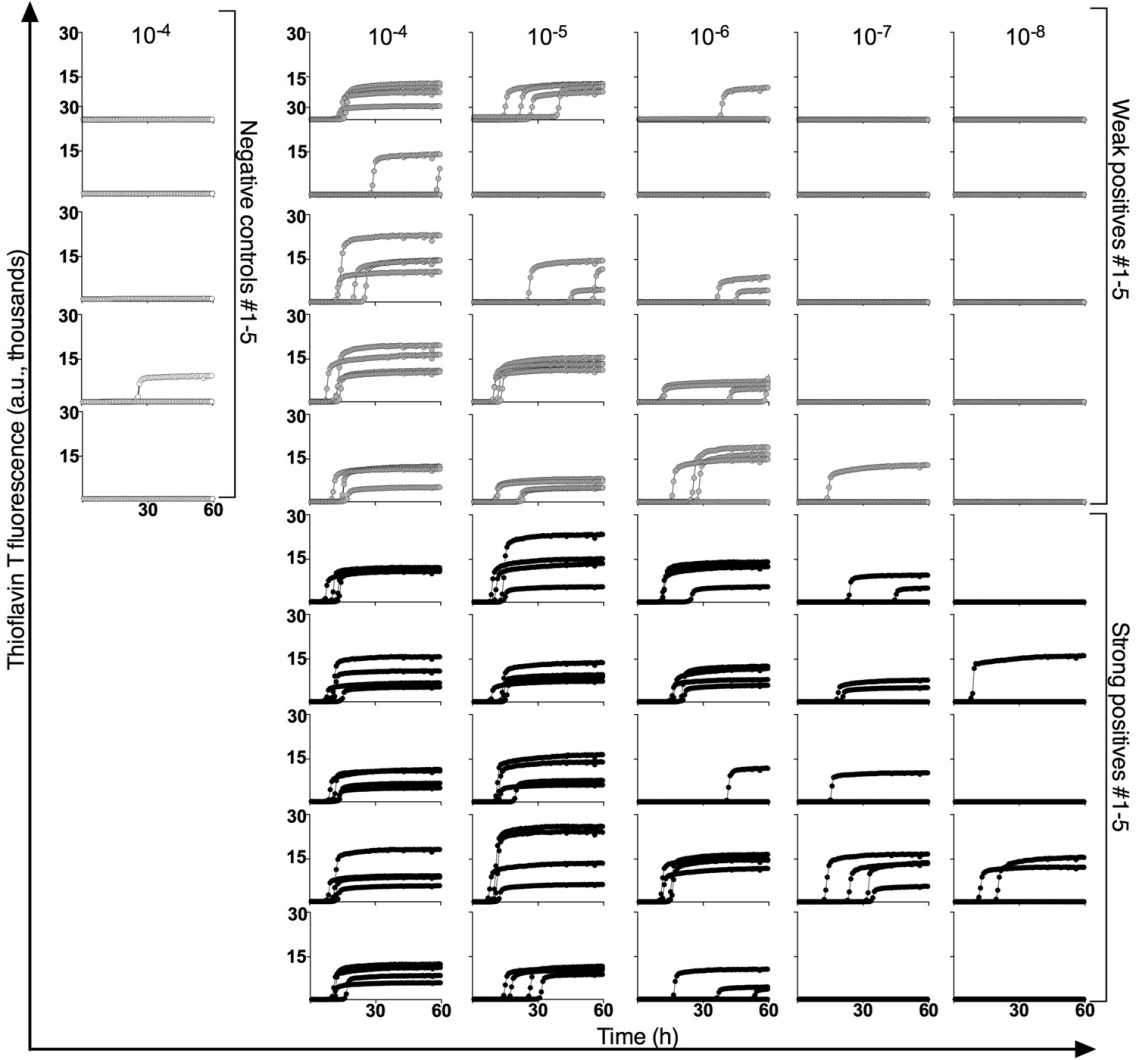
RT-QuIC reactions seeded with RLN homogenates from deer with RLNs that gave either negative, relatively weak, or strong ELISA readings as described in the main text. Replicate aliquots (n = 4) of the designated dilution of each homogenate were independently assayed in microplate wells with the thioflavin T fluorescence indicated in arbitrary relative units (a.u.) as a function of reaction time. Reactions seeded with 10^–4^ RLN tissue dilutions (w/v) from negative control deer (n = 5) or the designated dilutions from weak ELISA-positive (n = 5) and strong ELISA-positive deer (n = 5).

Analysis of postmortem RLN tissue for PrP^Sc^ by ELISA is an approved regulatory assay for identifying CWD-infected animals, as longitudinal studies have indicated that mule deer can become positive 3–6 months after oral inoculation, well prior to the onset of symptoms^10^. Based upon the optical density (OD) value obtained through ELISA of one of their RLNs, deer were classified as negative (OD < 0.1), weak positive (0.1 < OD < 1.0), and strong positive (OD > 3.0). Figure 1 shows that, consistent with previous studies^28^, prion seeding activity can be detected in the other RLNs collected from deer with weak (WP) as well as strong (SP) ELISA-positive RLN samples, but not in ELISA-negative controls (N1–N5). End point dilution RT-QuIC analysis of RLN from the weaker ELISA positive deer (OD = 0.539) had seed concentration that was ~ 100-fold lower than the deer with the strong ELISA positive RLN (OD = 3.999). Analysis of RLNs from a total of ten ELISA-positive deer indicated that the RLNs from weak positive deer (n = 5) had lower (p = 0.04, two tailed *t* test) mean (± SD) log 50% seeding units (doses)^15^, i.e. log SD_50_, per mg tissue (5.75 ± 0.91) than those from strong-positive deer (7.00 ± 0.64 log SD_50_ per mg; n = 5).

### IOME enhances the sensitivity of the RT-QuIC assay seeded with ear pinna homogenates

**Figure 2:**
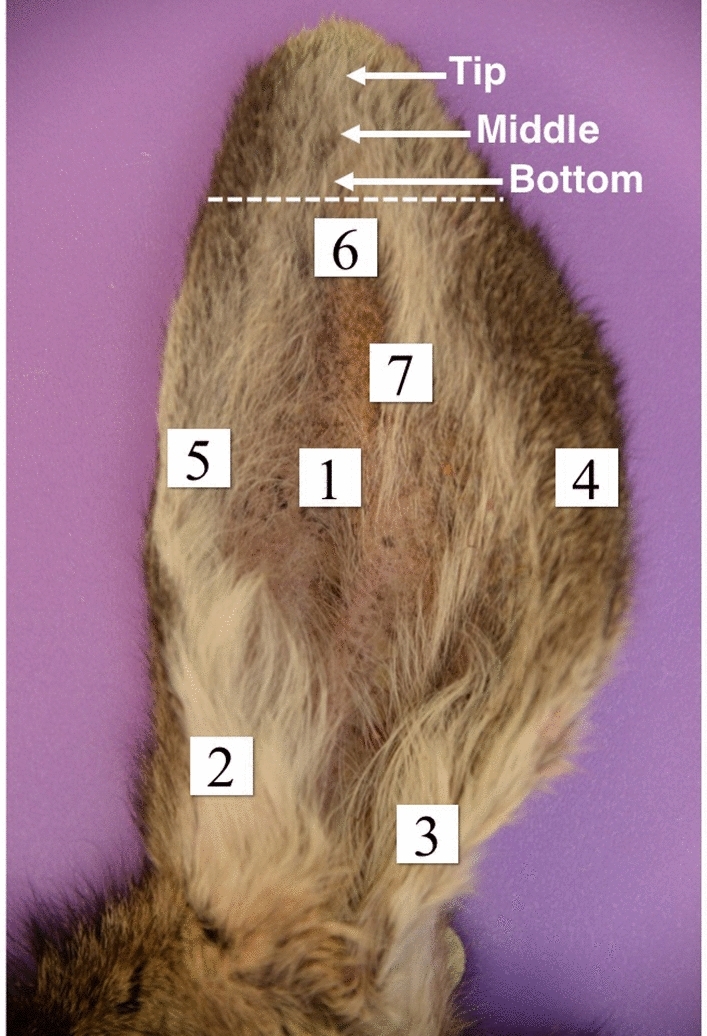
Representative picture of the ear parts from which the skin tissue was collected. The region of the apex of the ear above the dashed line shows areas samples for the initial survey of ear specimens using the original, suboptimal CWD RT-QuIC assay conditions. The numbers below the dashed line indicate areas from which 0.5 cm punches were taken for the analysis shown in Fig. 4.

**Figure 3:**
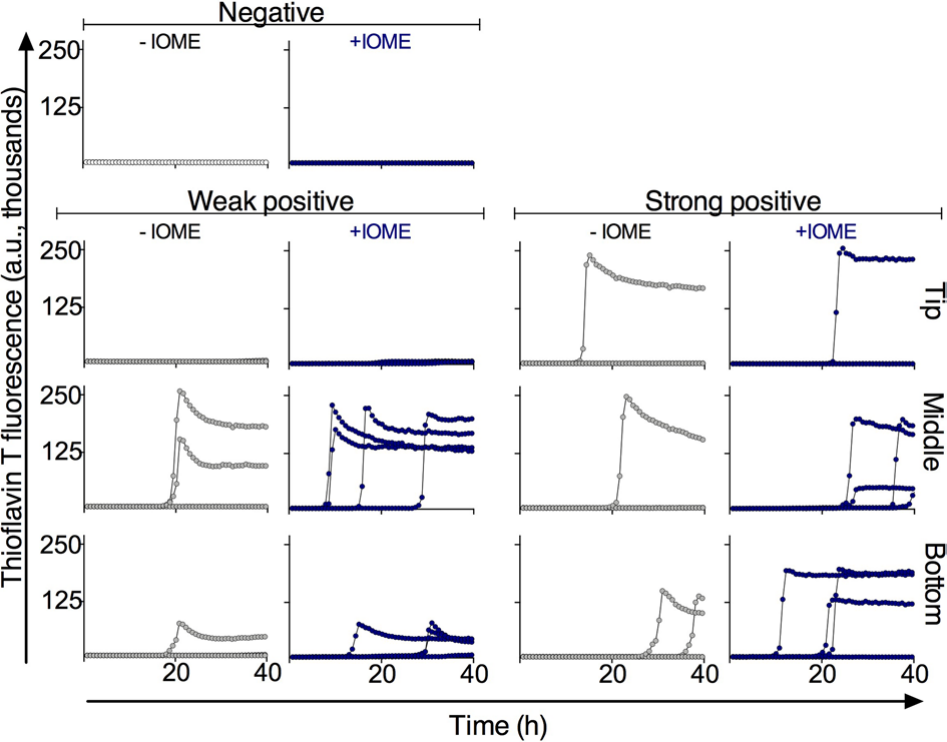
Fluorescence traces from individual wells (n = 4 per sample) of RT-QuIC reactions seeded with homogenates of ear punches collected from different regions of pinna apex (above the dashed line in Fig. 2). The RT-QuIC reactions (without IOME extraction, − IOME) were seeded with 10^–2^ dilution of ear homogenate in 0.1% SDS/PBS/N2. For the IOME-RT-QuIC reactions, a 1:10 dilution of iron oxide magnetic beads in 0.1% SDS/PBS/N2 was prepared, and the reactions were seeded with ear tissue equivalents comparable to the 10^–2^ dilution used in the reactions without IOME. ‘Weak positive’ and ‘strong positive’ labels refer to the ELISA positivity of RLN tissue from the same deer.

Aiming to develop an assay that would allow the use of a tissue sample that is more accessible than RLN or obex, we assessed the ability of RT-QuIC to detect prion seeding activity in post-mortem ear pinna punches from infected deer. We first used our previous CWD-adapted RT-QuIC assay to analyze a panel of 167 apparently asymptomatic hunter-killed deer that were designated as apparent CWD-positive and -negative deer according to ELISA assays of their RLN. However the diagnostic sensitivity provided by that assay was insufficient (< 50%). Therefore, to improve sensitivity, we added NaI in the RT-QuIC reactions based on previous findings^29^ and an initial iron oxide magnetic extraction (IOME) step that has been shown previously to aid in prion seed concentration and removal of potentially interfering tissue matrix components^7^. IOME extraction exploits the tendency of prion to bind to certain metals. Punches from tip, middle, and bottom of the ear apex (Fig. 2) were assayed by RT-QuIC, preceded or not by IOME. As shown in Fig. 3, the IOME-coupled RT-QuIC (IOME-RT-QuIC) provided more positive replicate reactions using samples from deer with both weakly and strongly ELISA-positive RLNs. Thus, we employed IOME-RT-QuIC in subsequent tests.

### Higher prion seeding activity is found around the central nerve in the ear pinna

**Figure 4:**
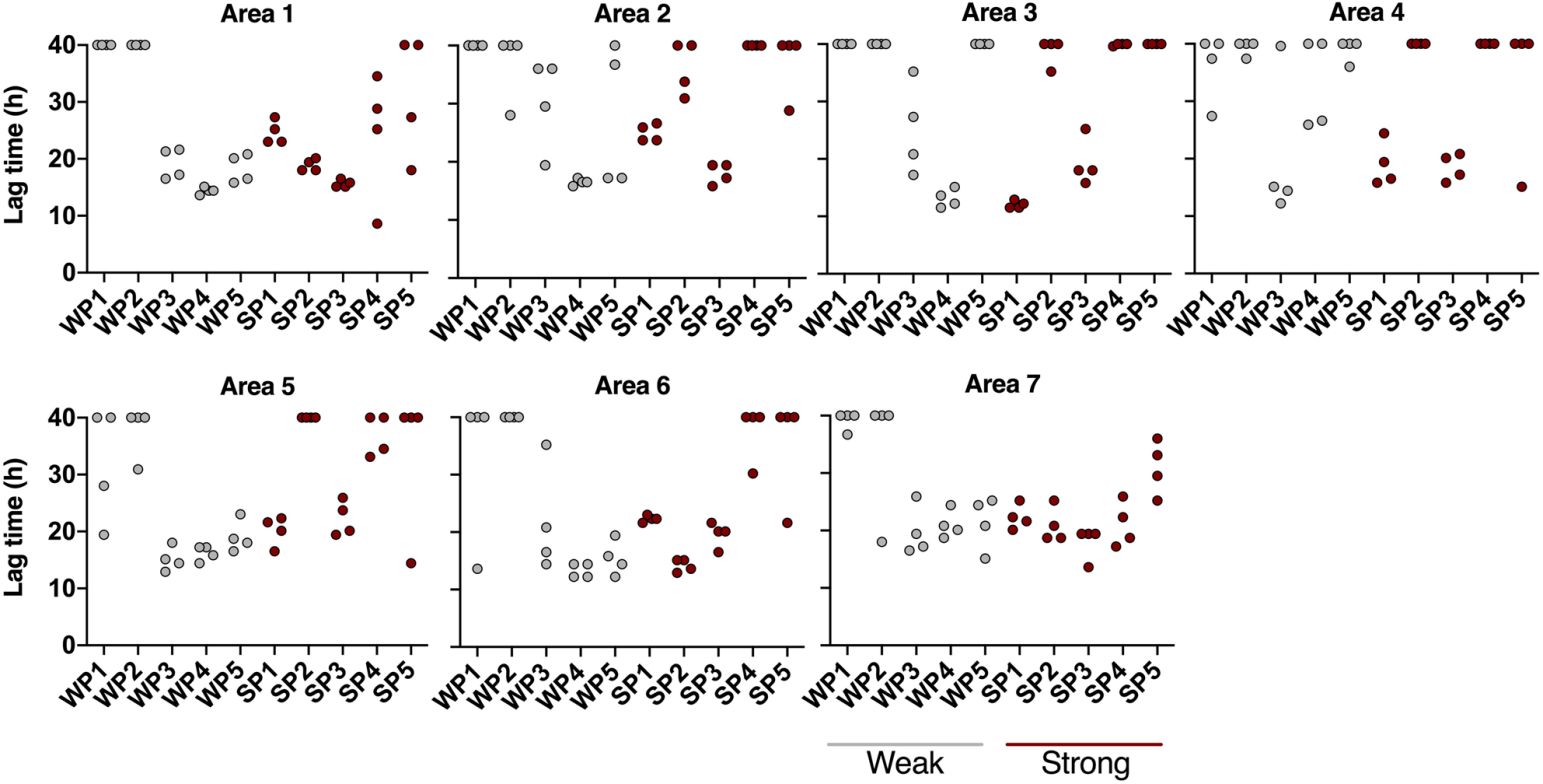
Lag times of RT-QuIC reactions seeded with ear pinna homogenates prepared from different areas (see Fig. 2) of five weak ELISA-positive (WP, grey) and five strong positive (SP, maroon) deer. Samples were subjected to IOME-RT-QuIC. Lag time (time to reach 10% maximum ThT fluorescence on the plate) within the cutoff time of the assay (40 h). Reactions with lag times of ≥ 40 h are aligned at the top of each graph.

Considering that prion seeding activity might not be evenly distributed throughout the ear pinna, we analyzed punches from seven different pinna areas as shown in Fig. 2 from 5 each of weak (WP) and strong (SP) ELISA-positive deer. After homogenization, samples were subjected to IOME-RT-QuIC. We found that prion seeding activity was more concentrated in areas 1, 6, and 7, which are located at (area 7) or adjacent to (1 and 6) the auricular nerve, with area 7 giving the highest proportion of positive reactions (Fig. 4).

### IOME-RT-QuIC analysis of a panel of ear pinna samples

Based on these results, we focused on sampling area 7 in a subsequent analysis of 58 ears collected from deer killed by hunters (n = 56) or submitted for diagnostic examination (n = 2). None of the hunter-killed deer had been reported as appearing unhealthy. Deer were deemed positive or negative for CWD infection based on both ELISA and RT-QuIC (without IOME) assays of their RLNs. These analyses were 100% concordant giving a total of 26 CWD-positive deer in the panel (Table 1). IOME-RT-QuIC analyses of pinna area 7 were performed by an operator who was blinded to the CWD status of the donors. Using the criteria described in the Materials and Methods for calling the samples positive or negative overall, 24 of the ear samples were positive (Table 2). Twenty-one of those samples were from deer with positive RLNs, giving an apparent diagnostic sensitivity of 81% based on the RLN diagnosis. The 3 remaining IOME-RT-QuIC-positive ear samples were from the group of 32 total deer with negative RLNs, giving 29 true IOME-RT-QuIC negatives out 32, or an apparent diagnostic specificity of 91%.

**Table 1:** Comparison of RLN ELISA (optical density; OD) and RT-QuIC results with ear (area 7) IOME-RT-QuIC results.

Sample ID^#,^*	CWD status**	OD	RLN RT-QuIC***	EAR RT-QuIC***
3681	Positive	3.999	4/4	4/4
3806w	Positive	3.999	4/4	4/4
5685w	Positive	0.490	2/4	1/4 and 1/4
7424	Positive	3.999	4/4	1/4 and 4/4
0482	Positive	0.389	4/4	4/4
0504	Not detected	–	0/4	0/4
0902	Not detected	–	0/4	0/4
0913	Not detected	–	0/4	0/4
0924	Not detected	–	0/4	0/4
1042****	Positive	0.689	4/4	4/4
1403	Not detected	–	0/4	0/4
2136	Positive	0.372	4/4	4/4
3422w	Positive	0.315	4/4	3/4 and 4/4
0262****	Not detected	–	0/4	0/4
0623	Not detected	–	0/4	0/4
0881	Not detected	–	0/4	0/4
0903	Not detected	–	0/4	2/4 and 2/4
1010	Not detected	–	0/4	0/4
1194	Positive	2.468	4/4	4/4
1356	Not detected	–	0/4	0/4
1894	Not detected	–	0/4	0/4
1920	Not detected	–	0/4	0/4
1953	Not detected	–	0/4	1/4 and 3/4
2546	Not detected	–	0/4	1/4 and 1/4
2550	Not detected	–	0/4	0/4
2561	Not detected	–	0/4	0/4
2594	Not detected	–	0/4	0/4
2616	Not detected	–	0/4	0/4
2863	Not detected	–	0/4	0/4
2922	Not detected	–	0/4	0/4
3353	Positive	1.252	4/4	0/4
3386	Positive	3.999	4/4	4/4
3482	Not detected	–	0/4	0/4
3493	Positive	3.999	4/4	3/4 and 4/4
3810w	Positive	3.999	4/4	0/4
4624	Not detected	–	0/4	3/4 and 1/4
4716	Positive	2.137	4/4	4/4
4720	Positive	3.999	4/4	4/4
4753	Positive	3.999	4/4	4/4
5630	Positive	1.565	4/4	4/4
5652	Not detected	–	0/4	0/4
5755	Not detected	–	0/4	0/4
5770	Not detected	–	0/4	0/4
5781	Not detected	–	0/4	0/4
5803	Positive	0.539	4/4	1/4 and 3/4
5851	Positive	3.999	4/4	2/4 and 4/4
7376	Positive	2.371	4/4	2/4 and 2/4
7472w	Not detected	–	0/4	0/4
7542	Positive	3.999	4/4	4/4
7995	Not detected	–	0/4	0/4
8264	Not detected	–	0/4	0/4
8360	Positive	3.999	4/4	4/4
0390w	Positive	0.453	4/4	4/4
0456	Not detected	–	0/4	0/4
2556	Not detected	–	0/4	0/4
2582	Positive	0.604	4/4	0/4
3385	Positive	0.373	4/4	0/4
5285	Positive	3.333	4/4	4/4

*Sample numbers with a “w” suffix were from white-tailed deer; all others were from mule deer.

**Based on RLN ELISA and RT-QuIC results in 3rd and 4th columns, which were 100% concordant.

***Positive/total replicate reactions. Two fractions for the same sample indicate results from repeated assays in quadruplicate.

****Carcass submitted for diagnostic examination. Samples not marked came from apparently healthy hunter-killed deer.

**Table 2:** Summary of RLN ELISA, RLN RT-QuIC and ear (area 7) IOME-RT-QuIC results from 58 deer (53 mule and 5 white-tailed).

	Assay results		Concordance of ELISA and RT-QuIC of RLN	Concordance of ear positives with RLN	App.* sensitivity or ear vs RLN	Concordance of ear negatives with RLN	App.* specificity of ear vs RLN
	+	−					
RLN (ELISA)	26	32	100%				
RLN (RT-QuIC)	26	32					
Ear (RT-QuIC)	24	34		21/26	81%	29/32	91%

*Apparent; we use this adjective to indicate that these sensitivity and specificity values are based on analysis of RLN tissues, which might not be a completely accurate indication of the overall CWD infection status of the deer.

## Discussion

Given concerns about the growing distribution of CWD and uncertainty about the potential for transmission to non-cervid species, it remains critical to improve the practicality and accuracy of detection of infection in both wild and farmed populations. One key challenge is the simple and cost-effective collection of diagnostic specimens from both live and dead animals in the field. Often this is done under less-than-optimal circumstances by people who may lack specific training in the relevant anatomy and the prevention of sample cross-contamination. Cross contamination can be a significant concern when the samples are being tested using ultra-sensitive prion seed amplification assays such as RT-QuIC. The collection of diagnostic specimens from live cervids presents particular problems in that sample collection usually requires restraint. As RLN tissue cannot be biopsied practically, several studies have focused on RT-QuIC analysis of RAMALT that can be obtained by rectal biopsy of cervids by trained personnel once the animal is restrained. Although these studies have yielded promising results as noted above, we hoped in undertaking this current study that the outer ear of cervids might be a useful specimen for RT-QuIC analysis due to ease of collection.

Our results show that IOME-RT-QuIC testing of deer pinna punch samples can provide sensitivity (81%) and specificity (91%) relative to RLN-based diagnosis (Table 2) that was better than, or comparable to, those previously reported for RT-QuIC analysis of RAMALT biopsy tissue (69.8% and > 93.9%, respectively)^22^. We note that whereas this previous RAMALT study sampled deer depopulated from a CWD-contaminated farm, the majority of samples we analyzed came from apparently asymptomatic deer killed by hunters. In neither study was the duration of the infection in the CWD-positive animals clear, but this same uncertainty will apply to most, if not all, naturally exposed cervids. Further experimental infection studies will be required to ascertain how early prion seeding activity can be detected in cervid ear pinna tissue after infections with various doses by different routes of inoculation. It is not clear why we failed to detect prion seeding activity in the area 7 ear samples of all deer that were positive by RLN analysis. However, one possible explanation is that the primary mechanism of spread to the ear pinna is via anterograde spread through nerves from the central nervous system. Indeed, the fact that we more frequently obtained positive IOME-RT-QuIC reactions from parts of the ear (i.e. area 7) that span the auricular nerve compared to other areas is consistent with this possibility. Given that evidence of infection is often seen in the lymphoreticular system of deer prior to detection in nervous tissues^10^, it seems likely that prion seeds might often appear in the ear pinna well after infection of RLNs.

Clearly, from a diagnostic perspective, there is room for improvement in the diagnostic performance of IOME-RT-QuIC analysis of outer ear tissue relative to standard *postmortem* RLN analysis. Sensitivity might be improved by exploring new methods of sample handling and RT-QuIC testing, unless the false negativity rate that we obtained was due entirely to deer being infected without yet having any CWD prions in their outer ears. There may also exist other cutaneous sites that are amenable to punch biopsy and IOME-RT-QuIC analysis that may harbor higher doses of PrP^Sc^, beyond what is found in the ear pinna during pre-clinical CWD infection. The apparently less-than-perfect specificity of IOME-RT-QuIC might be due to inadvertent cross-contamination of the sample, or occasional non-CWD components of ear pinna tissue that accelerate spontaneous nucleation and fibrillization of our recombinant PrP substrate. Such confounding factors might be more effectively removed from samples by as yet unknown improvements in sample handling and prion seed capture. On the other hand, the possibility remains that apparent false positive tests of ear samples were actually real positives obtained from deer that were CWD-infected in a way that was not yet manifest in their RLNs. For example, rare cases of CNS infection without lymphoid involvement have been observed^3^. Such cases might not only be a manifestation of infection route, but also CWD strain. Again, such possibilities would have to be addressed by deliberate, controlled experimental infection studies. Hopefully such studies will be encouraged by our current findings.

## Methods

### Samples collection

No animal-use-protocol approval was required for this study. The samples analyzed herein were collected opportunistically from heads or carcasses of deer that had been killed by licensed hunters (n = 56) or submitted for postmortem examination after dying as a result of injury or illness (n = 2). Entire ears were collected in addition to RNLs and biological data (species, sex, approximate age). Fresh RLN tissues were screened for evidence of PrP^Sc^ by ELISA^30^ at the Colorado State University Veterinary Diagnostic Laboratory (Fort Collins, Colorado USA). Ears and remaining RLN tissue samples were stored frozen until assayed.

### Recombinant PrP

Recombinant prion protein was expressed and purified as described previously^15^. Briefly, Syrian golden hamster prion protein sequence encompassing residues 90-231 was inserted into the pET41 vector (EMD Biosciences) and then transformed into Rosetta (DE3) *E. coli*. Protein expression was induced by the autoinduction system^31,32^. Protein was purified from inclusion bodies in an AKTA fast protein liquid chromatographer (GE Healthcare Life Sciences) using nickel-nitrilotriacetic acid (NTA) Superflow resin (Qiagen). The refolding process was performed under a Gdn-HCl reduction gradient and elution was achieved with an imidazole gradient. Protein was then dialyzed against 10 mM Na_2_HPO_4_ buffer (pH 5.8).

### Skin homogenization

The tissue was sprayed with 70% ethanol and then shaved with a scalpel. The shaved area was washed with PBS and the tissue was cut in 0.5 cm square pieces. The small pieces of tissue were placed in a tube with 1 mm glass beads, weighed, and homogenization buffer (2 mM CaCl_2_, 2.5 mg/mL collagenase A [0.25% w/v] in PBS) was added in order to make a 10% ear homogenate. The samples were homogenized in a bead beater (max power for 3 min) and incubated overnight at 37 °C in a thermomixer under mild agitation (650 rpm). Samples were agitated in a bead beater again (max power for 1 min), centrifuged for 5 min at 2000*g* to remove gross debris, and the supernatant was transferred into a new tube. Samples were stored at − 80 °C.

### Retropharyngeal lymph nodes homogenization

Retropharyngeal lymph nodes (RLN) were homogenized following the same protocol described above, except for the omission of shaving and washing with ethanol.

### Iron oxide magnetic extraction (IOME) coupled with RT-QuIC analysis

The protocol described here was developed based on a previously described IOME method^7^ with a couple of modifications. Superparamagnetic iron oxide beads (~ 9 μm, Bangs Laboratories, Indiana) were vortexed and 10 μL were transferred into a tube and washed with 0.5 mL phosphate buffered saline (PBS). After washing, beads were transferred to a tube containing 0.5 mL 0.1% SDS/PBS/N2 and 0.5 mL of 10% ear homogenate. The sample was incubated at room temperature in an end-over-end rotator for 2 h. Then, the beads were separated from the supernatant by placing the tube in the magnetic particle separator. Supernatant was removed and beads were resuspended in 10 μL of 0.1% SDS/PBS/N2. The suspension was sonicated for 1 min and a tenfold dilution of IOM beads was done in a 0.1% SDS/PBS/N2 solution. Two μL of this final dilution were added to single wells of a 96-well plate previously loaded with 98 μL of the RT-QuIC reaction mix (300 mM NaI, 10 mM Hepes, 1 mM EDTA [ethylenediamine tetraacetic acid], 10 μM thioflavin T, 0.1 mg/mL rPrP hamster 90-231). Each sample was assessed in quadruplicate, and following the first analyses, every sample which showed partial positive reactions (1–3 out of 4) was reanalyzed. A sample was considered positive when at least half of total replicates surpassed the threshold (10% maximum ThT fluorescence on the plate). Determination of log SD_50_ values by end-point dilution RT-QuIC analysis was performed as described previously^15^.
